# Determinants and Consequences of Limited Health Literacy in Polish Society

**DOI:** 10.3390/ijerph17020642

**Published:** 2020-01-19

**Authors:** Mariusz Duplaga

**Affiliations:** Department of Health Promotion and e-Health, Institute of Public Health, Faculty of Health Sciences, Jagiellonian University Medical College, Grzegorzecka Str. 20, 31-531 Krakow, Poland; mariusz.duplaga@uj.edu.pl

**Keywords:** health literacy, telephone-based survey, health behaviours, utilisation of health care, representative sample

## Abstract

Background: Health literacy (HL) is perceived as one of the most important concepts for modern health promotion activities to be successful. The research undertaken in the context of HL usually focuses on its antecedents and consequences, either for specific groups of patients or society or for the whole population. Objectives: The main aim of this study was to assess the antecedents and consequences of limited health literacy (HL) in a nationally representative sample of the Polish population. Methods: The analysis was carried out on the data obtained from a sample of 1000 Polish citizens through a telephone-based survey undertaken using a short, 16-item questionnaire developed within the European Health Literacy Project (HLS-EU). The total HLS score was calculated according to the guidelines published by the HLS-EU project. Chi2 test and logistic regression models were used for the analysis of the relationships between the variables. Results: The mean HL score (standard deviation) in the study sample was 12.99 (3.11). HL was related to age, marital and vocational status. Limited HL was associated with a lower self-assessment of health (OR, 95% CI: 2.52, 1.54–4.13), the prevalence of obesity and disability (1.71, 1.13–2.57, and 1.92, 1.25–2.94, respectively), less frequent physical activity (0.70, 0.49–0.99), a lower consumption of fruits and vegetables (0.47, 0.34–0.65), and with more frequent hospitalisations (2.02, 1.38–2.95). Conclusions: The assessment of HL using the16-item HLS-EU questionnaire may be a useful tool to enable health behaviours and utilisation of health care resources by society to be predicted.

## 1. Introduction

Health literacy (HL) is perceived as one of the most important concepts for modern health promotion activities to be successful. In the Declaration arising from the 9th Global Conference of Health Promotion held in 2016 in Shanghai, health literacy was indicated as a critical determinant of health [1]. The Declaration reinforced the message about HL being a pivotal tool to empower citizens and enable them to engage with collective health promotion actions [1]. In the available literature, there are many definitions of health literacy. The one which is often cited originates from the “Health Promotion Glossary” published in 1998 under the auspices of the World Health Organisation (WHO), which defines HL as “the cognitive and social skills which determine the motivation and ability of individuals to gain access, understand and use information in ways which promote and maintain good health” [2]. A comprehensive review of the existing HL definitions and models was carried as part of the European Health Literacy Project (HLS-EU) [3]. Apart from a detailed literature search, the researchers from the project developed an integrated model of health literacy encompassing access, understanding, appraisal, and application of health-related information in three domains; health promotion, disease prevention, and health care [3]. 

The research undertaken in the context of HL usually focuses on its antecedents and consequences, either for specific groups of patients or citizens or for the whole population. The available evidence from population studies shows that health literacy may depend on sex, age, level of education, economic and/or social status, and the type of vocational activities. In the research carried out to date, it has been demonstrated that lower HL is displayed by men rather than by women [4,5,6,7], by people having lower levels of educational attainment than by those with higher levels [4,5,6,7,8,9,10,11,12], by single people than by married people [7,13], by people with lower social status than by those with a higher status [8,11,14], by people of lower rather than those of higher economic status or income [4,7,8,9,13,15,16] and, finally, by people living in more challenging conditions [6,8]. In most studies, there was also a general trend for HL decreasing with age [4,6,8,9,15]. 

The efforts undertaken in the last decade resulted in a comprehensive assessment of the consequences of limited health literacy. In 2011, the updated version of the systematic review authored by Berkman et al. [16] was published. This stated that low HL might be associated with more frequent hospital admissions and the use of emergency care; lower participation in screening programmes, e.g., mammography; lower receipt of influenza vaccine; as well as a lower appreciation and understanding of health information, e.g., that provided on labels attached to health products. It was also found that in older groups, low HL may be associated with lower overall health status and with higher mortality rates [16]. More recent reviews have confirmed, or have revealed new findings about, the consequences of low HL in various groups of patients and citizens. For example, according to Humphry et al. [17], low HL may be associated with poor uptake of cancer screening, difficulty in making treatment choices and reduced quality of life following a diagnosis of cancer. In turn, the review of Zaben and Khalil [18] showed that low HL in patients with acute coronary syndrome is associated with their reduced quality of life. 

During the HLS-EU project, the consequences of low HL were thoroughly assessed [4]. The European survey was carried out using a 47-item questionnaire developed by the project (HLS-EU-Q47) [19] in eight European countries. It revealed that limited health literacy was associated with poor health status, having more than one long-term illness and the higher use of health care services involving six or more consultations with doctors in the last 12 months [4]. Other studies, in which national versions of the HL questionnaire developed within the HLS-EU project were used, confirmed that low HL may be associated with people’s poor self-assessment of health status [12,20], the prevalence of long-term illnesses [12], the higher utilisation of health care resources [14,21], and a lower level of physical activity [22,23,24].

Although the scope of information which arises from the HLS-EU-Q47 is extensive, it appears that the size of the tool may result in reduced compliance of respondents, especially if the questionnaire is used as a part of a survey which is also focused on other issues. Therefore, a shortened but also validated, the 16-item version has been used in many surveys (HLS-EU-Q16) [5,12,25,26,27,28,29]. The HLS-EU-Q16 questionnaire has been used not only in direct interviews with respondents [27,29] but also for telephone-based studies [12,26,30] or online surveys [31]. In some studies, the paper-based questionnaire was self-administered by respondents [32]. 

The only previous study of HL on a nationally representative sample of the Polish population was undertaken as part of the HLS-EU project [4] using the HLS-EU-Q47 questionnaire. This survey was carried employing a computer-assisted personal interview (CAPI) technique in July and August 2011 on a sample of 921 respondents. The mean general HL score in Poland was 34.45, which was lower than that in the Netherlands and Ireland and broadly the same as in Germany. Of the Polish respondents, 10.2% possessed inadequate general HL and 34.4% problematic HL. The general HL score showed a low correlation with age, education level, the main employment status, and the self-assessed social status but a moderate correlation with the self-assessment of financial deprivation. It would appear that no follow-up surveys are available. The main aim of this study is to report on the assessment of health literacy using the HLS EU-Q16 tool as well as undertaking an analysis of the possible antecedents and consequences of limited HL in the Polish population. 

## 2. Materials and Methods 

### 2.1. Survey

The analysis reported in this paper was based on the data originating from the survey carried out on a nationally representative sample of respondents at least 18 years old (*n* = 1000) in December 2016. The participants of the survey were recruited by a third party, Biostat Company (Biostat Sp. z o.o., Rybnik, Poland) [33], a company which has considerable experience in the conduct of opinion polls. This minimum size of the sample was established after taking into consideration the size of the population (31,535,606, according to Statistics Poland, the central statistical office in Poland [34]), the fraction of 0.5, and a confidence level of 0.95. For the sample of 1000 respondents, the level of the sampling error was 3.1%. A technique of computer-assisted telephone interviewing (CATI) was used in the survey. It was carried out by the CATI System’s research panel of interviewers employed in the Biostat Company [33]. The selection of the respondents relied on stratified proportional sampling from a database of mobile and stationary phone numbers developed by the Company. The structure of the sample corresponded to the Polish population relating to age, education, place of residence and NUTS1 regions. The strata were based on data given in the 2015 Local Data Bank of Statistics Poland [34].

The study received the consent from the Bioethical Committee of Jagiellonian University (No. 122.6120.313.2016 issued 24 November 2016).

The questionnaire used in the survey consisted of 58 items and included the HLS-EU-Q16 (Polish version of items applied in the survey performed initially within the HLS-EU project), an 8-item Polish version of eHEALS scale (Pl-eHEALS) [35,36], and a set of items asking about the use of Internet-based health information, health behaviours, self-assessment of health status, chronic diseases, disability status, and attitudes toward the possibility of public health interventions. Items exploring a series of key socio-demographic factors were also included in the integrated questionnaire. 

### 2.2. Health Literacy Score

The respondents could assign one of four responses to items in the HLS-EU-Q16 (very difficult, fairly difficult, fairly easy, very easy). In the event of them not being able to select, or they did not wish to select, any of these options, the interviewer was supposed to mark the particular item “difficult to say/not applicable”. The score based on the responses to the 16 items was calculated according to the recommendations of the HLS-EU project team [37]. The response options, “very difficult” and “fairly difficult”, were assigned with the score 0 and response options “fairly easy” and “very easy”—with the score 1. The response “difficult to say/not applicable” was regarded as a missing value. The total score was calculated as a sum of the subscores obtained for the individual items, but only if the number of missing values was not greater than 2. The evaluation of the16-item version of the HLS-EU questionnaire confirmed its adequate reliability; the Cronbach’s alpha coefficient was 0.902, and the Guttman half-split coefficient was 0.820. 

As recommended by Pelikan et al. [37], based on the total score, three categories of HL were established: “inadequate” for a score below 9, “problematic” for a score in the range from 9 to 12, and “sufficient” for a score above 12. In this reported study, the first two categories have been combined into a “limited HL” category as in the study reported by Levin-Zamir et al. [27].

### 2.3. Antecedents and Consequences

Following a review of literature, it was decided to include the sociodemographic variables including sex, age, education level, place of residence, marital status, professional activity as well as the use of the Internet, the use of mobile telephony and the time spent watching TV in the analysis as antecedents of HL. It was also assumed that HL might be associated with the self-perception of health status, the presence of chronic disease or disability, the prevalence of obesity, health behaviours and the utilisation of health care, this being based on the use of health care services and hospital admissions in the preceding 12 months.

### 2.4. Statistical Analysis

Statistical analysis was performed with the IBM SPSS v.24 software (IBM Corp. Armonk, NY, USA). Descriptive statistics were calculated for variables used in the analysis; absolute and relative frequencies for categorical variables and the mean and standard deviation (SD) for continuous variables. In the analysis, the chi2 independence test and univariate and multivariate logistic regression models were used. The level of *p* < 0.05 was treated as being significant. 

In the first step, the association of the variables reflecting the potential antecedents or consequences with HL was evaluated with the chi2 test. As a second step, the association of the potential determinants of HL was assessed with univariate logistic regression models. The effect of limited HL on potential outcome variables was assessed further by employing multivariate logistic regression models after making an adjustment for sex, age, and the level of education of respondents. 

### 2.5. Logistic Regression Modelling

Before the multivariate logistic regression models were developed, the multicollinearity was assessed. The variance inflation factor and the tolerance were in the expected ranges for all three models. For each multivariate regression model, the Hosmer and Lemeshow chi2 test and the Nagelkerke R square were calculated. For the independent variables included in logistic regression models, the p value, the odds ratio (OR) and 95% confidence interval (95% CI) are reported.

## 3. Results

### 3.1. The Characteristic of the Study Group

The mean age (SD) of the respondents participating in the survey was 45.9 (16.2) years of age. In the study group (*n* = 1000), 52.3% were women. Of the respondents, 28.3% lived in rural areas, 32.7% resided in urban areas with a population at least 100,000. 56.4% of the survey group declared an education level below upper secondary and 38.5% possessed a university bachelor’s or master’s degree. Of the respondents, 58.0% were married, singles comprised 29.0% and widowed, divorced or persons in separation 13%. Of the surveyed persons, 54.6% were employed in the public or private sector or were self-employed (entrepreneurs or farmers), 28.0% were retired, or on a disability pension, 8.4% were University or high school students, and the remaining 9.0% were vocationally inactive. In the study group, 84.9% were users of the Internet, and a total of 92.6% were mobile telephone users, including 64.2% who owned a smartphone. 

### 3.2. The Distribution of the Responses to HLS-EU-Q16 

The percentage of missing values due to the responses “difficult to say/not applicable” ranged from 1.3% for item 4 to 11.6% for item 12 on the questionnaire, as shown in Table 1. The response “fairly easy” was selected by more than 50% of respondents for all items, but “very difficult” was selected least frequently with the highest percentage being 3.9% for item 12. 

An HLS-EU-Q16 score could be determined for 84.2% (*n* = 842) of the respondents. The mean score (SD) was 12.99 (3.11), with a median of 14.00. After dichotomisation of the score, of the 842 for whom a score could be determined, 34.8% (*n* = 293) respondents were categorised with a limited HL, and 65.2% (*n* = 549) with sufficient HL. 

### 3.3. Antecedents

The analysis of the potential antecedents of HL carried out with the chi2 test showed a statistically significant association with the marital and vocational status (Table 2). Further analysis based on the univariate logistic regression revealed that there was a significant association between HL and age, marital and vocational status (Table 2). Specifically, respondents 50–59 years old were less prone to have limited HL than those aged 18–20 years (OR, 95% CI: 0.61, 0.38–0.98). Furthermore, married persons less frequently had limited HL than singles (OR, 95% CI: 0.65, 0.47–0.89). Finally, students and pupils were 1.8 times more likely to have limited HL than respondents who were employees or self-employed (OR, 95% CI: 1.80, 1.08–3.00). 

### 3.4. Consequences

The chi2 test revealed that HL was associated with the self-assessment of health status (*p* = 0.001), disability status (*p* = 0.006), the prevalence of obesity (*p* = 0.019), the intensity of physical activity (*p* = 0.048), the consumption of fruits and vegetables *(p* < 0.001), the consumption of fast food (*p* = 0.022) and hospital admission in preceding years (*p* < 0.001) (Table 3). The multivariate logistic regression models, in which the effects of dichotomised HL variable were adjusted for sex, age, and education level, confirmed most of these associations. The persons with limited HL, 2.5 times more frequently (OR, 95% Cl: 2.52, 1.54–4.13), those with disability nearly twice as often (OR, 95% CI: 1.92, 1.25–2.94) and obese persons 1.7 times more frequently (OR, 95% CI: 1.71, 1.13–2.57), assessed their health status as unsatisfactory than those with sufficient HL. 

Furthermore, such respondents less frequently undertook any form of physical activity in the preceding month (OR, 95% CI: 0.70, 0.49–0.99) or consumed fruits and vegetables (OR, 95% CI: 0.47, 0.34–0.65). Finally, these respondents were 2 times more often admitted to hospital in last the 12 months (OR, 95% CI: 2.02, 1.38–2.95). 

## 4. Discussion

In this paper, the results of the analysis of the possible determinants of HL, as well as the association of limited HL with the self-assessment of health status, the prevalence of a chronic disease or/and disability, health behaviours and the utilisation of health care resources, are reported. The paper reports the first assessment of the HL of Polish society carried out five years after the HLS-EU survey. For this assessment, a short, 16-item version of the HLS-EU questionnaire was used. On the basis of the responses to this questionnaire, an HLS-EU-Q16 score was calculated according to the HLS-EU project team’s recommendations. It was possible to calculate the score for 84.2% of the respondents to the questionnaire. The score was dichotomised into categories of “limited” and “sufficient” HL. Further analysis was carried out from the perspective of the antecedents and consequences of the possession of limited HL. 

For those for whom a score could be calculated, it was found that 34.8% had limited HL. A very similar level of inadequate and problematic HL was found by Levin-Zamir et al. [27] in a national sample of respondents in Israel. These researches also undertook their survey using the HLS-EU-Q16 tool but adapted to Hebrew, Russian, and Arabic. In 2013–2014 the “German Health Update” study was carried out using the HLS-EU-Q16 questionnaire and showed that inadequate and problematic HL was as high as 44.2% [26]. Tiler et al. [25] reported the results of the assessment of HL in 1,107 urban elderly adults from Eastern Germany recruited for the 2013 wave of the CARLA study. Although these authors used the HLS-EU-Q16, they calculated HL scores using the method applied earlier for the HLS-EU-Q47 giving results in four categories of HL. Therefore, their results are not fully comparable with the results of the Polish survey. Nonetheless, it is relevant to report that in their study, the frequency of inadequate HL was 4%, and that of problematic HL was 23%.

The frequency of limited HL in Polish society was much higher than among respondents from Catalonia as reported in a recent study by Garcia-Codina et al. [29]. They performed a survey of HL on a group of 2433 inhabitants [27]. The total frequency of respondents with inadequate and problematic HL was only 15.4%. However, the validation study of the HLS-EU-Q16 carried out on 223 Italian respondents from Florence and its surroundings published by Lorini et al. [12] stated as many as 67% of the respondents displayed inadequate or problematic HL. 

In two studies undertaken in African countries, the frequency of limited HL (inadequate or problematic) was much higher than has usually been reported in the studies carried out on European populations. Almaleh et al. [7] used the adapted HLS-EU-Q16 tool to assess the HL of patients attending an outpatient clinic of one of the University hospitals. In this sample, only 18.9% of respondents demonstrated sufficient HL. The frequency of inadequate HL was 34.3% and of problematic HL 46.7%. In turn, the study performed by Amoah [28] among inhabitants of the Ashanti Region in Ghana revealed that the frequency of inadequate HL was 24.0% and of problematic HL 38.8%.

The HLS-EU-Q16 instrument was applied by Wangdahl et al. [38] in the survey performed in 2015 on a group of 455 adult refugees in Sweden. In this group, only 38.2% of the respondents had sufficient HL. A high frequency of inadequate and problematic HL measured with the HLS-EU Q16 tool on a group of Somali refugee women in Oslo was reported by Gele et al. [39]. It should be noted that these authors calculated the total score and established categories of HL analogically, as did Tiler et al. [25].

The analysis of possible antecedents of HL in the Polish population showed statistically significant association only with age, marital and vocational status. Limited HL occurred less frequently among the respondents aged 50–59 years than among those aged 18–29, similarly less frequently among married persons than singles and finally, among employed or self-employed than among students and pupils. 

Jordan and Hoebel [26] reported in 2015 that HL measured with the HLS-EU-Q16 tool was associated with educational level but not with the sex and age of respondents. In the study of Tiler et al. [25], there was a positive association between HL and age, educational level, net household income, and self-perceived social position. Interestingly, in this study, women displayed a lower HL than men. 

The study of Levin-Zamir et al. [27] found, that after controlling for other determinants only the number of years of education and the level of income were significantly associated with HL. Garcia-Codina et al. [29] reported that low health literacy was associated with lower levels of education and low socioeconomic status. These researchers regarded a physical limitation that restricted the ability to perform everyday activities as an antecedent of HL and confirmed that it was strongly associated with low HL (OR, 95% CI: 2.50, 1.34–4.66). In turn, Lorini et al. [12] showed that the HLS-EU-Q16 score (as assessed with the chi2 independence test) was associated with the level of education and being trained or employed in healthcare. In this study, the prevalence of long-term illness, treated as an antecedent, was also associated with low HL. In the Egyptian population, inadequate HL was more frequently found in males and in persons with a low level of education [7]. The study of Amoah [28] in Ghana showed that HL was associated with age, place of residence, marital status, education level and income. Recently, Eronen et al. [40] carried out face-to-face interviews with a group of 292 Finns aged 75 years old in order to assess their HL. The authors used the HLS-EU-Q16 tool but calculated the HL score using the method recommended for the HLS-EU-Q47 by the HLS-EU project team [40]. These authors found that the HL, from all analysed sociodemographic and economic variables, was associated only with the perceived financial situation. Those who assessed their situation as very good demonstrated the highest HL.

The reported pattern of relationships between sociodemographic factors and the level of HL is different in the various studies. However, the most persistent finding in other studies is a significant association between a person’s HL and their level of education. Interestingly, such a relationship was not found in our study. However, the HLS-EU project team reported a low but statistically significant correlation for the Polish population [4]. However, their analysis was based on the survey using the CAPI technique and the use of the standard 47-item HLS-EU questionnaire. It should be recognised that in Polish schools, the curriculum does not focus on the development of health-related knowledge or skills. The lack of any association between the level of education and HL should strengthen the current efforts to include health education in Polish school curricula.

As for the consequences, applied multivariate logistic regression models revealed that limited HL, after adjustment for sex, age, and education level, was associated with a lower self-assessment of health status, the prevalence of obesity and disability, less frequently undertaking of physical activity and the lower consumption of fruits and vegetables. Respondents with limited HL had also more frequently been admitted to hospital in the preceding year.

The findings of the Polish survey are in line with the results reported by Jordan and Hoebel [27]. They found that higher HL was associated with beneficial health behaviours and that low HL was related to poorer physical and mental health. Tiler et al. [25] reported that there was an inverse association between HL and the prevalence of myocardial infarction among women, diabetes in both sexes and strokes in men. Levin-Zamir et al. [27] also used logistic regression to analyse the relation between HL and self-assessment of health status and with health behaviours, including sun protection, smoking and physical activity. However, the association of HL was statistically significant only with the self-assessment of health. 

In a study involving 9617 members of a Belgian health insurance fund it was confirmed that low HL was associated with more admissions to one-day clinics, general practitioner home consultations, psychiatric consultations, ambulance transportation and with longer stays in general hospitals [31]. The study undertaken by Garcia-Codina et al. [29] showed that low HL was modestly associated with low levels of physical activity, having self-perceived chronic disorders and not undertaking preventive activities. According to Lorini et al. [12], the HL score calculated from HLS-EU-Q16, was related only with self-perceived health status, and not with the BMI category, doctor’s visits, emergency department admissions, admissions to hospital or access to outpatient specialist care. The study of Amoah [28] on the inhabitants of one region in Ghana confirmed that the level of HL enabled the prediction of health status and wellbeing. Finally, Eronen et al. [40] using the Spearman correlation coefficient, demonstrated that lower HL was associated with lower cognitive status and self-assessment of health, more frequent depressive symptoms and chronic conditions, lower life-space mobility, and physical performance. The association between low HL determined by the HLS-EU-Q16 score and unfavourable health behaviours was also reported for inhabitants of rural areas in Indonesia by Mubarokah [41]. 

There are many other papers reporting the results of the evaluation of the potential determinants and consequences of limited HL, measured with other versions of the HLS-EU questionnaire, and with other types of instruments. Nonetheless, the discussion presented here has been focused on the results reported by recent studies using the HLS-EU-Q16 tool. 

The importance of HL is not fully appreciated in Poland. Apart from the activities of the HLS-EU project completed in 2012 [4], there have been no significant attempts to assess the HL of the general population or specific groups of respondents. There are only a few review papers focused on HL available in Polish literature. Furthermore, HL has never been included as a target or an indicator in the initiatives undertaken within the public health domain even in the current National Health Programme for 2016–2020 [42]. It was also not addressed in the Law on Public Health issued in 2015 [43].

The results of the current study can have important implications for the provision of health services and public health activities in Poland. Firstly, it appears that about 35% of the general population has limited HL and the high percentage may be an indicator of an inadequate level of health education being provided by the educational system. It may also reflect on the relative weakness of health communications addressed to society. The analysis of potential antecedents showed that young adults, especially pupils or students, and singles, may be at risk of limited HL. These observations justify the recent initiative to introduce a health education programme to the school curriculum in 2021 and justify the reorientation of information strategies employed in health care facilities. To date, older patients have been perceived as the group that requires special attention when providing medical advice and explanation. It seems that health professionals should be advised to provide clearer communications when interacting with young adults. Our research revealed that limited HL is associated with obesity. Such a link supports the inclusion of broader initiatives and interventions to enhance HL to counteract the growing trend towards an overweight and obese society. Furthermore, in health programmes targeting unfavourable health behaviours, e.g. concerning nutritional habits or physical activity, one of the critical objectives should be the development of adequate HL.

Interestingly, our study showed also that there is a statistically significant relationship between limited health literacy and hospital admissions. One might expect that hospital admission should be associated with higher HL. However, it is also probable that persons with low HL may be prone to readmissions because they are unable to self-manage their long-term illnesses, and therefore, they are at risk of disease exacerbations. The promotion of the concept of HL-friendly health institutions, including the screening for patients with limited HL, and implementing strategies to enhance the HL of such patients, could be an appropriate initiative to avoid unnecessary hospitalisations.

### Limitations

There are several aspects of this study that need to be considered. Firstly, the use of the HLS-EU-Q16 tool is in itself related to certain limitations in the evaluation of potential antecedents and consequences of limited HL. It should be noted that the arbitrary classification of the resulting HLS score to limited and sufficient HL groups may be related to the lower sensitivity in detecting interrelations with the variables characterising the respondents. In this reported study, the CATI technique was applied. Although more and more researchers go beyond the initial technique of direct interviews, it is not clear how the use of CATI or online survey influences the sensitivity of the HLS-EU tools. With appropriate instruction being given to interviewers who connect with the respondents, a lack of response resulting in a missing value could be only assigned if the respondent had a real problem with giving a response. Even with such an approach, the HLS-EU-Q16 score could not be calculated for nearly 16% of the respondents. Finally, the use of the HLS-EU-Q16 tool and the CATI technique does not allow for a full comparison of the obtained results with the first survey assessing HL in Polish society which was undertaken as part of the HLS-EU project. 

## 5. Conclusions

The HL categories established on the basis of the score originating from the response to the 16-item version of the HLS-EU questionnaire are relatively insensitive to options assumed by sociodemographic variables. Among the variables treated in the study as the antecedents of HL, significant associations were confirmed only for age, marital status, and vocational status. However, it was possible to confirm that there were statistically significant relationships between HL and several variables modelled as consequences, including the self-assessment of health, the prevalence of obesity and disability, the intensity of physical activity, the consumption of fruits and vegetables and the number of admissions to hospital in the preceding year. 

The undertaken analysis reported in this paper showed that limited HL is associated with a less favourable self-assessment of health status, the prevalence of obesity and disability, less favourable health behaviours and making use of health care resources such as hospital admissions more frequently.

## Figures and Tables

**Table 1:** The distribution of responses to individual items included in the European Health Literacy Project (HLS-EU)-Q16 *.

HLS-EU-Q16 Item	Difficult to Say/Not Applicable %	Very Difficult %	Fairly Difficult %	Fairly Easy %	Very Easy %
1. Find information on treatments of illnesses that concern you?	7.5	1.4	16.8	51.7	22.6
2. Find out where to get professional help when you are ill?	2.9	0.9	14.2	57.5	24.5
3. Understand what your doctor says to you?	3.5	0.9	9.2	62.9	23.5
4. Understand your doctor’s or pharmacist’s instruction on how to take a prescribed medicine?	1.3	0.8	4.2	60.1	33.6
5. Judge when you may need to get a second opinion from another doctor?	8.0	1.6	23.2	53.0	14.2
6. Use information the doctor gives you to make decisions about your illness?	6.9	1.1	14.8	62.5	14.7
7. Follow instructions from your doctor or pharmacist?	3.0	0.7	9.3	59.0	28.0
8. Find information on how to manage mental health problems like stress or depression?	8.5	2	18.5	54.1	16.9
9. Understand health warnings about behaviour such as smoking, low physical activity and drinking too much?	3.2	1.1	7.6	52.4	35.7
10. Understand why you need health screenings?	9.8	2.1	13.7	53.2	21.2
11. Judge if the information on health risks in the media is reliable?	9.6	2.8	22.0	52.6	13.0
12. Decide how you can protect yourself from illness based on information in the media?	11.6	3.9	20.9	52.3	11.3
13. Find out about activities that are good for your mental well-being?	10.3	2.2	18.8	54.7	14.0
14. Understand advice on health from family members or friends?	5.5	1.3	10.5	61.3	21.4
15. Understand information in the media on how to get healthier?	9.2	2.1	14.7	57.7	16.3
16. Judge which everyday behaviour is related to your health?	5.0	1.4	12.9	58.6	22.1

* Only relative frequencies (%) were provided in the table due to the fact that total sample *n* = 1000.

**Table 2:** The assessment of potential antecedents of limited health literacy (HL) with chi2 test and univariate logistic regression.

Independent Variable	Categories of an Independent Variable	Health Literacy		*p* Value ^a^	OR	95% CI	*p* Value ^b^
		Limited % (*n*)	Sufficient % (*n*)				
Sex	Male *	34.6 (136)	65.4 (257)	0.913	1		
	Female	35.0 (157)	65.0 (292)		1.016	0.77–1.35	0.913
Age	18–29 *	39.5 (66)	60.5 (101)	0.317	1		
	20–39	37.1 (66)	62.9 (112)		0.90	0.58–1.39	0.641
	40–49	33.8 (48)	66.2 (94)		0.78	0.49–1.25	0.30
	50–59	28.4 (40)	71.6 (101)		0.61	0.38–0.98	0.041
	≥60	34.1 (73)	65.9 (141)		0.79	0.52–1.21	0.792
Education level	lower than upper secondary *	33.8 (49)	66.2 (96)	0.644	1		
	upper secondary or post-secondary non-tertiary	35.3 (125)	64.6 (228)		1.07	0.72–1.61	0.73
	University degree	34.6 (119)	65.4 (225)		1.04	0.69–1.56	0.87
Place of residence	Rural *	33.3 (79)	66.7 (158)		1		
	urban <20,000	30.8 (36)	69.2 (81)	0.937	0.89	0.55–1.43	0.629
	urban from 20,000 to <100,000	36.3 (77)	63.7 (135)		1.14	0.77–1.68	0.507
	urban from 100,000	36.6 (101)	63.4 (175)		1.15	0.80–1.66	0.44
Marital status	Single *	41.3 (102)	58.7 (145)	0.024	1		
	widowed or divorced or in separation	36.3 (37)	63.7 (65)		0.81	0.50–1.30	0.384
	married	31.2 (154)	68.8 (339)		0.65	0.47–0.89	0.007
Household net income	≤1500 PLN *	34.1 (70)	65.9 (135)	0.586	1		
	1500–2500 PLN	32.8 (63)	67.2 (129)		0.94	0.62–1.43	0.778
	>2500 PLN	37.0 (128)	63.0 (218)		1.13	0.79–1.63	0.501
Vocational status	employee or self-employed *	35.0 (165)	65.0 (306)	0.040	1		
	retired or on disability pension	31.7 (71)	68.3 (153)		0.86	0.61–1.21	0.386
	university or school student	49.3 (34)	50.7 (35)		1.80	1.08–3.00	0.023
	unemployed	29.5 (23)	70.5 (55)		0.78	0.46–1.31	0.34
Internet use	No *	38.9 (44)	61.1 (69)	0.321	1		
	Yes	34.2 (249)	65.8 (480)		0.81	0.54–1.22	0.321
The use of mobile telephony	non-user *	33.3 (19)	66.7 (38)	0.965	1		
	mobile phone but not smartphone	35.2 (81)	64.8 (149)		1.09	0.59–2.01	0.79
	smartphone	34.8 (193)	65.2 (362)		1.07	0.60–1.90	0.828
Watching TV	≤1 h daily *	33.5 (73)	66.5 (145)	0.768	1		
	>1 h daily	34.6 (211)	65.4 (399)		1.05	0.76–1.46	0.77

^a^*p* for chi^2^ independence test, ^b^ *p* for univariate logistic regression with HL as a dependent variable (limited HL vs sufficient HL), * referential categories in the logistic regression models for limited HL.

**Table 3:** The analysis of potential consequences of limited HL (chi2 independence test and multivariate logistic regression models adjusted for sex, age, and education level).

Dependent Variable	Categories of a Dependent Variable	Health Literacy		*p* Value ^a^	OR	95% CI	*p* Value ^b^
		Limited % (*n*)	Sufficient % (*n*)				
Self-assessment of health status	At least satisfactory *	33.1 (253)	66.9 (512)		1		
	Unsatisfactory	51.9 (40)	48.1 937)	0.001	2.52	1.54–4.13	<0.001
Chronic disease	No *	32.5 (233)	67.5 (484)		1		
	At least one	46.2 (48)	53.8 956)	0.396	1.24	0.91–1.68	0.168
Disability	No *	32.5 (233)	67.5 (484)		1		
	Yes	46.2 (48)	53.8 (560	0.006	1.92	1.25–2.94	0.003
Obesity	No (BMI < 30.0) *	33.3 (243)	66.7 (487)		1		
	Yes (BMI ≥ 30.0)	44.6 (50)	55.4 (62)	0.019	1.71	1.13–2.57	0.010
Tobacco smoking	Non-smoker *	33.8 (133)	66.2 (260)		1		
	Active or passed smoker	35.1 (153)	64.9 (283)	0.706	1.09	0.82–1.47	0.551
Alcohol consumption	No consumption in last month *	33.9 (81)	66.1 (158)		1		
	At least once in last month	34.8 (196)	65.2 (367)	0.802	1.01	0.72–1.41	0.971
Physical activity	No physical activity *	41.2 (73)	58.8 (104)		1		
	At least once in last month	33.2 (200)	66.8 (403)	0.048	0.70	0.49–0.99	0.042
Consumption of fruits and vegetables	Less often than once daily *	47.4 (118)	52.6 (131)		1		
	At least once daily	29.4 (173)	70.6 (416)	<0.001	0.47	0.34–0.65	<0.001
Fast food consumption	Not more than once in last month *	32.9 (183)	67.1 (374)		1		
	More often than once on last month	43.0 (64)	57.0 (85)	0.022	1.44	0.97–2.12	0.071
Use of health care services in the last 12 months	No use *	29.1 (37)	70.9 (90)		1		
	At least once	35.6 (251)	64.4 (454)	0.158	1.40	0.92–2.13	0.113
Hospital admission in last 12 months	No admission *	32.3 (229)	67.7 (481)		1		
	At least once	48.5 (64)	51.5 (68)	<0.001	2.02	1.38–2.95	<0.001

^a^ p for chi^2^ independence test, ^b^ p for multivariate logistic regression with HL as an independent variable (sufficient HL used as a reference category for limited HL), * referential categories in the logistic regression models for limited HL.
